# Expression, Docking, and Molecular Dynamics of Endo-*β*-1,4-xylanase I Gene of* Trichoderma virens* in* Pichia stipitis*

**DOI:** 10.1155/2017/4658584

**Published:** 2017-08-10

**Authors:** Gammadde Hewa Ishan Maduka Wickramasinghe, Pilimathalawe Panditharathna Attanayake Mudiyanselage Samith Indika Rathnayake, Naduviladath Vishvanath Chandrasekharan, Mahindagoda Siril Samantha Weerasinghe, Ravindra Lakshman Chundananda Wijesundera, Wijepurage Sandhya Sulochana Wijesundera

**Affiliations:** ^1^Department of Chemistry, Faculty of Science, University of Colombo, Colombo, Sri Lanka; ^2^Department of Plant Sciences, Faculty of Science, University of Colombo, Colombo, Sri Lanka; ^3^Department of Biochemistry and Molecular Biology, Faculty of Medicine, University of Colombo, Kynsey Road, Colombo 08, Sri Lanka

## Abstract

It is essential that major carbohydrate polymers in the lignocellulosic biomass are converted into fermentable sugars for the economical production of energy. Xylan, the major component of hemicelluloses, is the second most naturally abundant carbohydrate polymer comprising 20–40% of the total biomass. Endoxylanase (EXN) hydrolyzes xylan into mixtures of xylooligosaccharides. The objective of this study was to genetically modify* Pichia stipitis*, a pentose sugar fermenting yeast species, to hydrolyze xylan into xylooligosaccharides via cloning and heterologous extracellular expression of* EXN*I gene from locally isolated* Trichoderma virens* species.* Pichia stipitis* was engineered to carry the* EXN*I gene of* T. virens* using pGAPZ*α* expression vector. The open reading frame encodes 191 amino acids and SDS-PAGE analysis revealed a 24 kDA recombinant protein. The EXNI activity expressed by recombinant* P. stipitis* clone under standard conditions using 1% beechwood xylan was 31.7 U/ml. Molecular docking and molecular dynamics simulations were performed to investigate EXNI-xylan interactions. Free EXNI and xylan bound EXNI exhibited similar stabilities and structural behavior in aqueous medium. Furthermore, this in silico work opens avenues for the development of newer generation EXN proteins that can perform better and have enhanced catalytic activity.

## 1. Introduction

Production of alternate fuels is crucial for economic development and therefore second and third generation bioethanol production have become key areas of research in many countries [1, 2]. Lignocellulosic biomass consists of cellulose (40–60%), hemicellulose (20–40%), and lignin (10–30%). Much research has already been focused on biofuel generation using cellulose. However, for economically feasible production of ethanol, the hemicellulose of plant biomass also should be utilized in an effective manner [3, 4].

Hemicelluloses are linear or branched heteropolysaccharides comprising different types of sugar residues, namely, xylose, mannose, galactose, arabinose, or glucose [5, 6]. The major component in hemicellulose is xylan, a polymer of 1,4-linked *β*-D-xylose units [7]. Xylanases, namely, *β*-1,4-xylosidases (XYL) and endo-xylanases (EXN), are responsible for depolymerization of xylan to xylose [8, 9]. Endo-*β*-1,4-xylanase (EXN) depolymerizes xylan to shorter xylobiose and xylooligomers [10] while *β*-1,4-xylosidases (XYL) hydrolyzes xylobiose and xylooligomers to D-xylose generated by the action of endo-xylanases (EXN). Isolation and purification of EXN enzymes from cellulolytic microorganisms have been strongly directed towards research and development due to its incomparable commercial applications such as in paper pulping and biobleaching and in food and animal feed industries [6, 11]. Among many different microbes, filamentous fungi including* Trichoderma* species have been identified as better xylanase producers and they have been used for more than 50 years in the production of industrial xylanase [12, 13].

Pentose fermentation pathway is restricted to a few yeast species such as* Pichia stipitis, Candida shehatae,* and* Pachysolen tannophilus* [14–16]. Therefore,* P. stipitis* has been genetically modified to improve xylose like pentose fermentation [17, 18]. The present study describes the characterization, cloning, and expression of* EXN*I from locally isolated* Trichoderma virens* species with the long-term objective of producing second generation biofuel from hemicellulose using recombinant* Pichia stipitis.* In this study, the pGAPZ*α* integrative vector consisting of yeast *α*-mating factor (MF*α*) signal peptide with a glyceraldehyde 3-phosphate dehydrogenase (GAP) promoter driven expression system was used.

A three-dimensional (3D) structure of EXNI was built using homology modeling with quality assessments for the understanding of the structural features and properties. A molecular docking study was carried out to investigate the docking of substrates to the catalytic site of the enzyme. Molecular dynamics (MD) simulations provide evidence for conformational changes in the molecular system and the time-dependent behavior of biomolecules and study how the enzyme functions are affected by xylan.

## 2. Materials and Methods

### 2.1. Culturing of* Trichoderma virens* and Xylanase Activity Assay

Locally isolated* Trichoderma* species was confirmed as* Trichoderma virens* using PCR based internal transcribed spacer (ITS) analysis [19]. The isolated species was cultivated for seven days on potato dextrose agar (PDA) at 30°C. An equal amount of inocula was selected from the above grown fungal culture using a sterile cork borer (5 mm diameter) and inoculated into conical flasks (100 mL) containing 25 mL of Mandel's medium (MM) (constituents of MM include (NH_4_)_2_SO_4_ 1.4 g/L, KH_2_PO_4_ 2.0 g/L, CaCl_2_ 0.3 g/L, MgSO_4_·7H_2_O 0.3 g/L, peptone 1.0 g/L, FeSO_4_·7H_2_O 5.0 mg/L, ZnSO_4_·7H_2_O 1.4 mg/L, MnSO_4_·H_2_O 1.6 mg/L, and CoCl_2_ 2.0 mg/L, pH 5.5) supplemented with 2% beechwood xylan [20, 21]. Cultures were incubated at 30°C, in a rotary shaker at 150 rpm. The enzyme extracts were harvested by filtration at 24 hour intervals for nine days. They were centrifuged at 6200*g* at 4°C for 10 minutes to obtain a cell free enzyme extract and stored at −20°C. Thereafter, the enzyme extracts were freeze dried and 100 *μ*L of the crude enzyme extracts was tested for endoxylanase (EXN) activity [22] using 900 *μ*L of 1% soluble beechwood xylan as the substrate. The reaction mixture was incubated at 50°C in a water bath for 5 minutes. Three milliliters of 3,5-dinitrosalicylic acid (DNS) solution was added to each reaction mixture and placed in a boiling water bath for 15 minutes for color development. The color intensity is proportional to the amount of reducing sugar (xylose) produced in the reaction mixture. Absorbance was measured at 540 nm to determine enzyme activity with reference to the substrate and enzyme blanks [23, 24]. One unit (1 U) of endoxylanase activity is defined as the amount of 1 *μ*mole of liberated hydrolysis product (equivalent to xylose) in 1 mL of enzyme volume per minute (*μ*mol m1^−1^ min^−1^). Enzyme activity was calculated with reference to the xylose standard graph.

### 2.2. Cloning and Sequence Analysis of* EXN1* Gene of* Trichoderma virens*

Gene specific primers were designed for PCR amplification of* EXNI* gene prior to cloning. Sequences homologous to* EXNI* were identified using the nucleotide blast search in NCBI. Open reading frame (ORF) of the consensus sequence of* EXNI* was determined using ORF finder (https://www.ncbi.nlm.nih.gov/orffinder/). The native signal sequence, predicted by the SignalP3.0 server (http://www.cbs.dtu.dk/services/SignalP/), was omitted in primer designing of* EXNI* to accommodate the *α*-mating factor signal sequence of the pGAPZ*α* vector.

Genomic DNA was extracted from* T. virens* using a simple extraction method [25] and* EXN1 *was amplified using the above designed primers. EXN1FP: 5′-ATCGTGAATTCCAGACGATTGGCCCCGGCACT-3′ and EXN1 RP: 5′-TTGATTCTAGATTAGCTYACGTTAATGTTGGCGTTACCAGAGCT-3′. PCR cycling conditions were optimized (initial denaturation at 94°C for 2 min followed by 35 cycles of denaturation at 94°C for 30 sec, annealing at 65°C for 30 sec, and extension at 72°C for 30 sec. This was followed by a final extension at 72°C for 7 min). The PCR product (~700) bp was electrophoresed on a 0.8% agarose gel, purified, and ligated into pGEM-T vector (Promega, USA). Thereafter, it was transformed into* E. coli* JM109 competent cells (Promega, USA) using the heat-shock method [26]. The transformants were spread on to low salt Luria-Bertani (LB) agar medium (1% tryptone, 0.5% yeast extract, 0.5% NaCl, and 1.5% agar, pH 7.5) containing ampicillin (100 *μ*g/mL), 5-bromo-4-chloro-indolyl-D-galactoside (0.2 mM), and isopropyl-thio-*β*-D-galactopyranoside (40 *μ*g/mL). Plasmid extractions were carried out on selected white colonies and custom sequenced (Macrogen, Korea). A recombinant* EXNI* clone was designated as pGEM-T/g*EXNI*. After sequence confirmation, pGEM-T/g*EXNI* was digested with* EcoRI* and* XbaI* restriction enzymes and the insert purified and ligated into pGAPZ*α* vector. Ligated products were transformed into* E. coli* JM109 and transformants were selected in zeocin (25 *μ*g/mL) containing low salt LB agar plates. The resulting clone was designated as pGAPZ*α*/g*EXNI*.

### 2.3. Transformation into* Pichia stipitis*

The pGAPZ*α*/g*EXNI* vector construct was linearized using* Bgl*II and purified. The plasmid concentration was determined and adjusted to ~1 *μ*g/*μ*L.* Pichia stipitis *(ATCC 58376) was inoculated into 0.5 mL YPD broth (1% yeast extract, 2% peptone, and 2% glucose) in a 1.5 mL microcentrifuge tube and incubated at 30°C overnight in a rotary shaker at 150 rpm. A volume of 500 *μ*L from the above grown culture was inoculated into 50 mL broth in a 250 mL conical flask and incubated at 30°C at 150 rpm until the OD_600_ reached 1.4. Yeast electrocompetent cells were prepared according to the procedure given in pGAPZ*α* vector manual (Invitrogen, USA). A volume of 80 *μ*L* P. stipitis* competent cells was mixed with 5–10 *μ*g of linearized pGAPZ*α*/g*EXNI *plasmid DNA. The mixture was subjected to electroporation under the optimized conditions of 1.5 Kw, 200 mA, and 25 *μ*F (pulse time of 5 ms) in a 0.2 cm electroporation cuvette. The resulting transformation mixture was spread on to YPDS (1% yeast extract, 2% peptone, 2% glucose, 2% agar, and 1 M sorbitol) plates with 100 *μ*g/mL zeocin as the selection marker. The plates were then incubated for 3 days at 30°C to obtain positive transformants. Twenty yeast colonies were selected and streaked on fresh YPDS plates containing zeocin (100 *μ*g/mL).

### 2.4. Screening of Recombinant Yeast

Colony PCR was performed for the above selected colonies to confirm the presence of the integrated* EXNI* gene in the* P. stipitis* genome. Native* P. stipitis* and pGAPZ*α*/*gEXNI* plasmid DNA were used as the negative and positive controls, respectively. PCR amplified product was subjected to agarose (0.8%) gel electrophoresis. A putative clone designated Y-pGAPZ*α*/g*EXNI* was sequenced. Thereafter it was cultured in YPD broth with 100 *μ*g/mL zeocin as the selection marker and incubated overnight. These cultures were used for expression analysis and long-term storage purposes in 50% glycerol at −80°C.

### 2.5. SDS-PAGE and Expression Analysis of* EXNI* in Recombinant* P. stipitis*

Y-pGAPZ*α*/*gEXNI* recombinant* P. stipitis* was inoculated into beechwood xylan broth culture and incubated overnight at 30°C in a rotary shaker at 200 rpm. Native* P. stipitis* was used as the control. The enzyme was harvested by centrifugation of culture medium at 12000 rpm for 2 min at 4°C. The enzyme extract was concentrated by freeze drying. SDS-PAGE analysis [27] was performed and the results were analyzed by comparing with native* P. stipitis*.

Enzyme activity assay was carried out on the Y-pGAPZ*α*/g*EXN* recombinant* P. stipitis* using native* P. stipitis* as the control. They were separately inoculated from YPD streak plates into 0.5 mL of YPD broth cultures in 1.5 mL microcentrifuge tubes and incubated for 3 days in a rotary shaker at 200 rpm at 30°C. The enzyme harvest was freeze dried and a volume of 900 *μ*L of solubilized 1% beechwood xylan was treated with 100 *μ*L of above enzyme extract. The standard assay procedure was performed and recombinant EXNI enzyme activity was quantitatively determined according to the method described in Section 2.1. Both enzyme and substrate controls were maintained throughout the assay procedure.

### 2.6. Structure Prediction and Evaluation of EXNI Protein

The tertiary structure of EXNI protein was generated with the aid of MODELLER (version 9.13) program [28, 29]. The most similar X-ray crystallographic structures were identified in RSCB PDB protein databank [30, 31] using the Blast Protein tool [32] against the deduced amino acid sequence of EXNI as the target. Multiple sequence alignment method was used for homology modeling and the generated model was based on the templates of PDB IDs: 2JIC, 4S2H, and 4HKW for EXNI in the RSCB PDB protein databank. The constructed model was validated using structure validation tools of VERIFY3D [33], PROCHECK [34], and ERRAT [35] to analyze the compatibility of the model with its amino acid sequence, to verify the geometrical and stereochemical constraints of the model and to determine the overall quality factor respectively. The COACH server (https://zhanglab.ccmb.med.umich.edu/COACH) [36, 37] was used to identify the binding domain of the above generated EXNI model.

### 2.7. Molecular Docking

The 3D structure of the oligoxylose ligand was constructed and geometrically optimized before docking, with 6-31g^*∗∗*^ basis set using Gaussian 09 (linux version) software [38]. Protein and above optimized ligand were prepared using UCSF chimera [39], and the ligand docked into the active site of the model structure of EXNI using DOCK6 software [40, 41] using flexible docking method. The binding strength of the protein and the ligand was ranked using the grid score energies [42].

### 2.8. Molecular Dynamics Simulation

Molecular dynamics simulation was performed in two phases. In the first phase, the docked protein-ligand complex obtained from above docking process with lowest binding energy was selected as the initial configuration for the molecular dynamics (MD) simulations using the GROMACS v4.6.5 [43]. The GROMOS54a7 united atom force field was assigned for the model protein. The force field parameters of the ligand were obtained from PRODRG server [44]. The protein-ligand complex was inserted in the center of a box with 9 × 9 × 9 nm^3^ volume. Electroneutrality of the system was maintained by adding Na^+^ ions to the box and solvated with SPC/E water molecules [45]. Electrostatic interactions were modeled by particle mesh Ewald (PME) with a short-range cutoff of 1.2 nm [46]. Berendsen's weak coupling algorithm was employed to maintain the temperature and pressure of the system at 300 K and 1 bar [47]. Using LINCS algorithm [48] all bonds were constrained at their equilibrium distances while allowing other internal motions of bending and torsion during molecular dynamics simulation. The system was subjected to 2000 steps of energy minimization with steepest descent algorithm followed by 200 ps long MD simulation to equilibrate the simulation system. After the equilibration step, 15 ns MD simulation was carried out using a desktop computer with Intel® Core™ i7-950 Processor. Configurations of the system at every 2 ps intervals were stored for further analysis. At the end of the simulation, the noncovalent interactions between ligand and the protein were analyzed by the LigPlot+v.14.5 software [49]. The same protocol was used in the second phase, to simulate the bare protein, obtained from protein homology modeling process.

The first phase molecular dynamics simulation was carried out to investigate the stability of the docked protein-ligand complex and the second phase molecular dynamics simulation was employed to investigate the structural comparison between docked complex and bare protein during the simulations in the aqueous medium.

## 3. Results and Discussion

**Figure 1:**
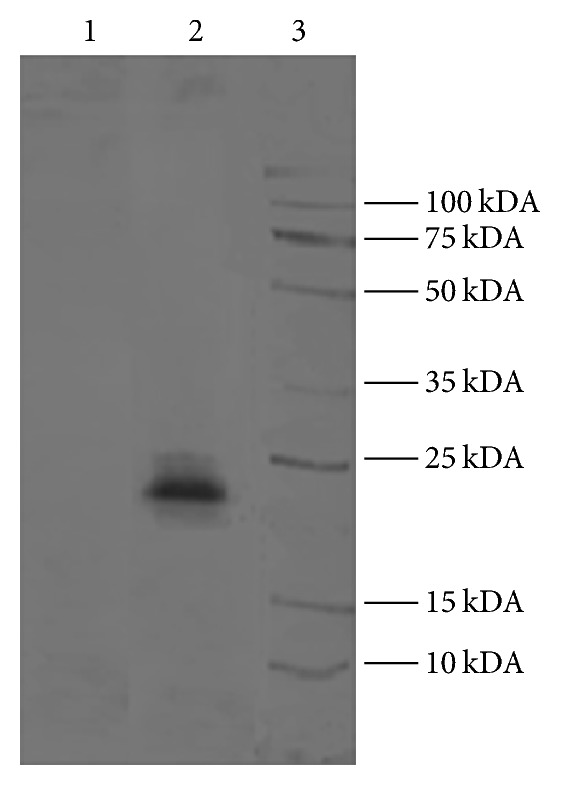
SDS-PAGE analysis of* P. stipitis* extracts. Lane (1): enzyme extract of nonrecombinant* P. stipitis*. Lane (2): recombinant EXNI enzyme (~24 kDA) secreted by recombinant Y-pGAPZ*α*/g*EXNI P. stipitis* clone. Lane (3): broad range protein molecular weight marker.

The maximum EXN activity of* Trichoderma virens* was 114.2 U/mL on day seven. PCR amplification of* EXN*I gene using the genomic DNA of* Trichoderma virens* yielded a 689 bp fragment (GeneBank: KJ882380). Sequence analysis revealed a single intron of 115 bp (from 274 bp to 387 bp). The coding region consists of a 573 bp open reading frame that encodes 191 amino acids. A sequence similarity search (NCBI) indicated that the above amplified* EXN*I was 99% identical to the DNA sequence of endo-1,4-beta-xylanase I gene of* Trichoderma asperellum* strain T-1 (KM277356.1) and 99% identical to the mRNA sequence of endo-1,4-beta-xylanase of* Trichoderma viride* strain YNUCC0183 (AY320048.1). Coding sequence of the* EXN*I was translated and was 100% similar to the* Trichoderma viride* amino acid sequence (AAP83925.1). Further, the amino acid residues ranging from 9 to 189 were identified to belong to the glycosyl hydrolases family 11 by the InterProScan server (EMBL). Many characterized xylanases are classified under this family. Three potential N-glycosylation sites at positions 61, 97, and 188 of the amino acid sequence were identified using NetOGlyc 4.0 Server and GlycoEP server. Search for O-glycosylation sites using the NetNGlyc 1.0 Server did not reveal any O-glycosylation sites. The theoretical molecular weight of the EXNI protein was calculated as 20.7 kDA and isoelectric pH was 8.75. SDS-PAGE analysis confirmed that the EXNI recombinant enzyme expressed by* P. stipitis* was ~24 kDA (Figure 1).

The EXNI enzyme activity expressed by Y-pGAPZ*α*/g*EXNI P. stipitis* clone was 31.7 U/mL indicating the successful approach of genetic engineering in the heterologous extracellular expression of EXNI using the (GAP) promoter driven expression system. However, the EXNI activity of recombinant Y-pGAPZ*α*/g*EXNI* was 3.6 times less than the native* T. virens*. It is reported that yeast species hyperglycosylated secretory recombinant proteins [50, 51]. The experimental molecular weight of the EXNI was comparatively higher (~24 kDA) than the molecular weight of the EXNI deduced from the amino acid sequence. There is thus a~3 kDA difference in the experimental and the theoretical molecular mass of EXNI. A possible explanation for the low enzyme activity could be due to hyperglycosylation of recombinant EXNI at N-glycosylation sites by* P. stipitis* compared to the native fungus. It has been reported that N-linked hyperglycosylation can have a significant effect on protein expression and function [52–54]. According to these studies N-linked hyperglycosylation usually occurs at the sequence Asn-Xaa-Ser/Thr (Xaa, any amino acid). As mentioned above, there are three potential glycosylation sites at 61 (Asn-Phe-Ser), 97 (Asn-Pro-Ser), and 188 (Asn-Val-Ser) which can be hyperglycosylated to support the above possibility. Recent mutational studies on glycosylation sites have confirmed that the presence of a proline residue between Asn and Ser/Thr will inhibit N-linked hyperglycosylation [55, 56]. At position 97 there is a proline residue that may inhibit hyperglycosylation. However, the other two positions are possible sites for hyperglycosylation.

The recombinant EXNI activity (31.7 U/mL) observed in the present study was considerably lower than the heterologous expression of different xylanases reported for* Pichia pastoris* in several recent studies where the highest activity ranged from 120 U/ml to 746 U/ml [8, 57–59]. These high enzyme activities are attributed to the low hyperglycosylation of recombinant proteins expressed by* P. pastoris* compared to expression of heterologous secretory proteins in other yeast species as the host [8, 60]. However,* P. pastoris* does not harbour pentose utilization enzymes needed for fermentation of xylose to ethanol. It is reported that the pentose utilization ability is much more efficient in* P. stipitis *compared to all other* Pichia* species [61–63]. To our knowledge, this is the only recorded study, for the heterologous expression of* Trichoderma* EXNI in* P. stipitis.* The long-term goal of the present study is to express both xylanases, EXN and XYL [64], in a yeast species to ferment xylan, the major component of hemicellulose in biomass. To achieve this goal the expression of recombinant EXNI in* P. stipitis* should be much higher than the present expression. This could be achieved by (i) introducing proline residues between Asn and Ser/Th or by introduction of specific amino acids, such as Trp, Asp, Glu, or Leu, residues between Asn and Ser/Thr at 61 and 188 positions of EXNI to convert the sequon to a poor oligosaccharide acceptor, to omit the hyperglycosylation. However, at the same time the integrity of the tertiary structure and the catalytic activity of the protein should be maintained with the aid of in silico strategies including homology modeling and molecular dynamics simulation techniques [65, 66]. (ii) According to the in silico strategies of this study the active site residues of EXNI were identified at positions 126–131. Random mutagenesis could be used to increase the acidity or the basicity of the above amino acid residues which can facilitate the catalytic activity under “retention” and “inversion” mechanisms described for the glycosyl hydrolases [67, 68]. (iii) An alternative approach would be to introduce genes encoding xylose utilizing pathway enzymes into* P. pastoris* by advanced genomic shuffling [69, 70]. This would involve recombination of entire genome of Y-pGAPZ*α*/g*EXNI* recombinant* P. stipitis* with that of* P. pastoris.*

Five probable models were obtained in homology modeling from the MODELLER 9.13 software and they were ranked according to their normalized Discrete Optimized Protein Energy (zDOPE) and GA341 score. The model comprising the best scores was selected as the theoretical model for EXNI protein.

According to the characterization of the EXNI model by DSSP program [71] the secondary structure of EXNI is composed of 1 *α*-helix and 14 *β*-sheets as given below.

*Alpha Helix*. VAL152-LEU162

*β-Sheets*. (GLY6-ASN10), (TYR13-ASN19), (VAL25-ASN29), (SER34-TRP39), (ASN44-TRP51), (VAL59-ASN69), (ASN71-SER80), (ILE85-PHE93), (THR103-SER110), (SER113-ARG122), (PH134-ARG141), (GLY148-THR151), (GLN172-TYR179), (SER182-SER190).

**Figure 2:**
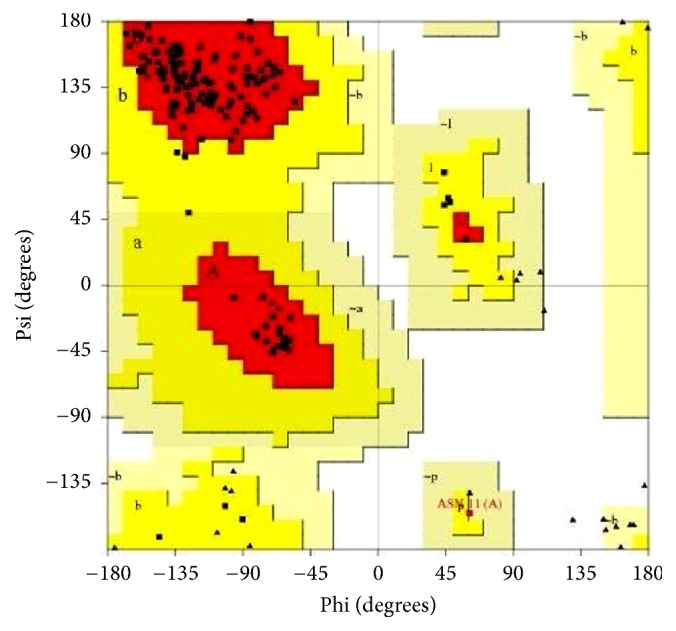
Ramachandran map of modeled EXNI protein.

Results from the PROCHECK analysis of EXNI are given in Table 1 and the Ramachandran plot generated by the same program is represented in Figure 2. According to the statistical score of the Ramachandran plot none of amino acids are in the disallowed region.

**Figure 3:**
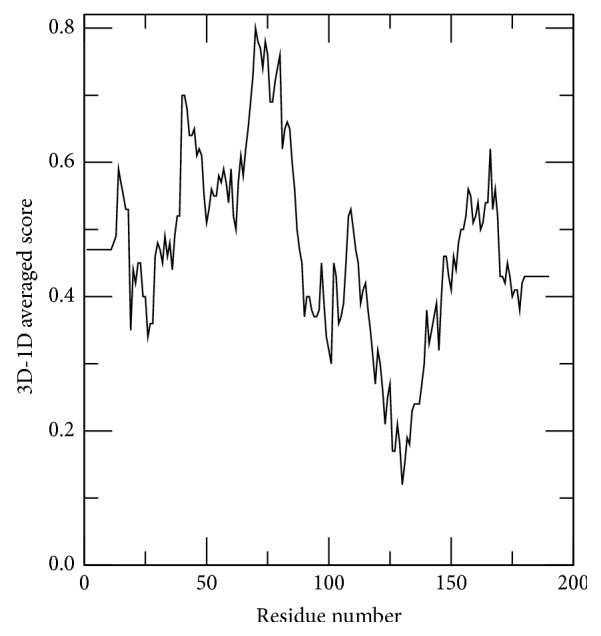
VERIFY3D score profile that shows more than 96.32% of residues having average 3D-1D score of greater than or equal to 0.2.

VERIFY3D profile of EXNI shows that all the residues have an averaged 3D-1D score greater than zero (Figure 3). Furthermore, to pass the VERIFY3D test, it is essential to show that at least 80% of the amino acids have scored more than 0.2 [33] in the 3D/1D profile. In this study, the results indicate that 96.32% of the residues had an averaged 3D-1D score of greater than or equal to 0.2. Moreover, the ERRAT program evaluated the overall quality factor as 86.81 for the modeled 3D structure of EXNI. Above evaluations concluded a highly reliable 3D structure of EXNI.

**Figure 4:**
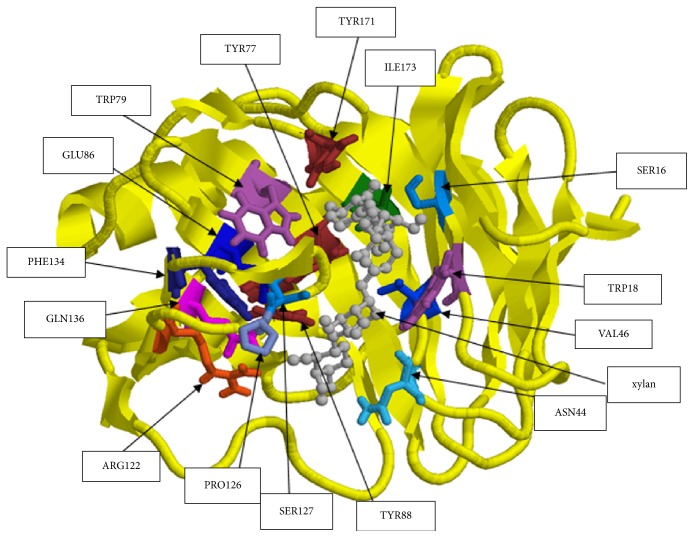
Protein-ligand complex from molecular docking.

The EXNI-xylan complex with the lowest binding energy was selected for the molecular docking method. Initially the binding residues of EXNI (TRP18, VAL46, TYR77, TRP79, GLU86, TYR88, ARG122, PRO126, SER127, PHE134, GLN136, TYR171, ASN44, TYR77, ILE173, and SER16) were identified using I-TASSER-COACH server. Above binding residues were interestingly matched with the results obtained from molecular docking. The recorded best grid score for EXNI-xylan was −80.47 kcal mol^−1^. Above grid score is a summation of van der Waals dispersive and electrostatic interaction energy which indicates the approximate binding energy of the ligand.  Figure 4 represents the best protein-ligand complex from molecular docking.

The general catalytic mechanisms of glycosyl hydrolases follow either retaining or inverting mechanisms with the assistance of acidic amino acid residues in the active site [67, 68]. According to the docking results the presence of glutamic and aspartic acids in the active site may perform any of the above mechanisms.

**Figure 5:**
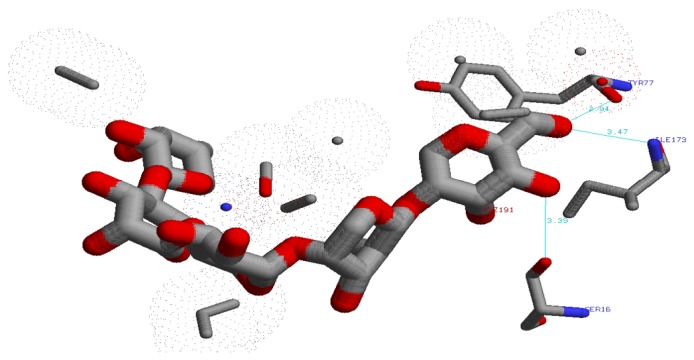
Three-dimensional view of H bonds between ligand and the protein residues.

**Figure 6:**
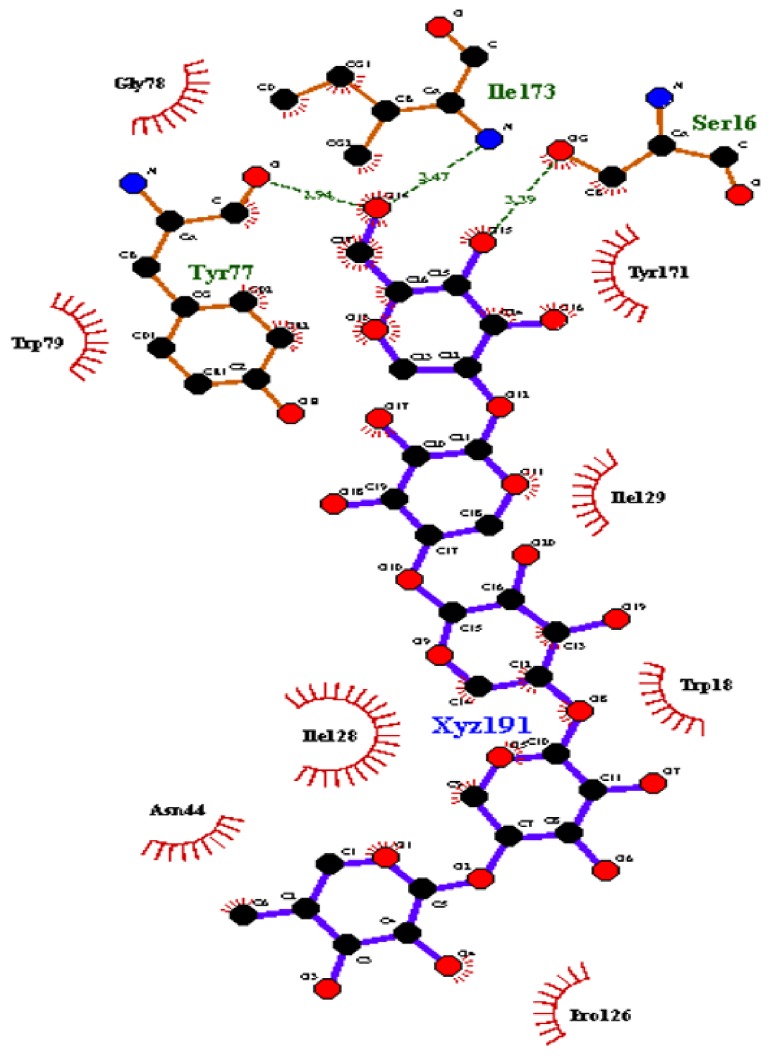
H bonds between ligand and the protein residues from LigPlot program.

Two MD simulations of 15 ns each were carried out for the protein-ligand complex and the bare protein in aqueous medium with 21923 SPC/E water molecules. The noncovalent interaction (H bond) of the final configuration (after 15 ns) of protein-ligand complex identified from LigPlot+v.145 software is presented in Figures 5 and 6. The LigPlot analysis of the protein structure indicates that the ligand forms three strong hydrogen bonds with SER16 (3.39 Å), ILE173 (3.47 Å), and TYR77 (2.94 Å). Detailed information is presented in Table 2. The stability of all these H bonds was studied using* g_dist* tool in the GROMACS program.

Throughout the simulation time the distance between the centers of mass of the two groups of atoms which was involved in H bond formation was maintained nearly at a constant value confirming the continuance, stability, and effectiveness of the H bonding.

**Figure 7:**
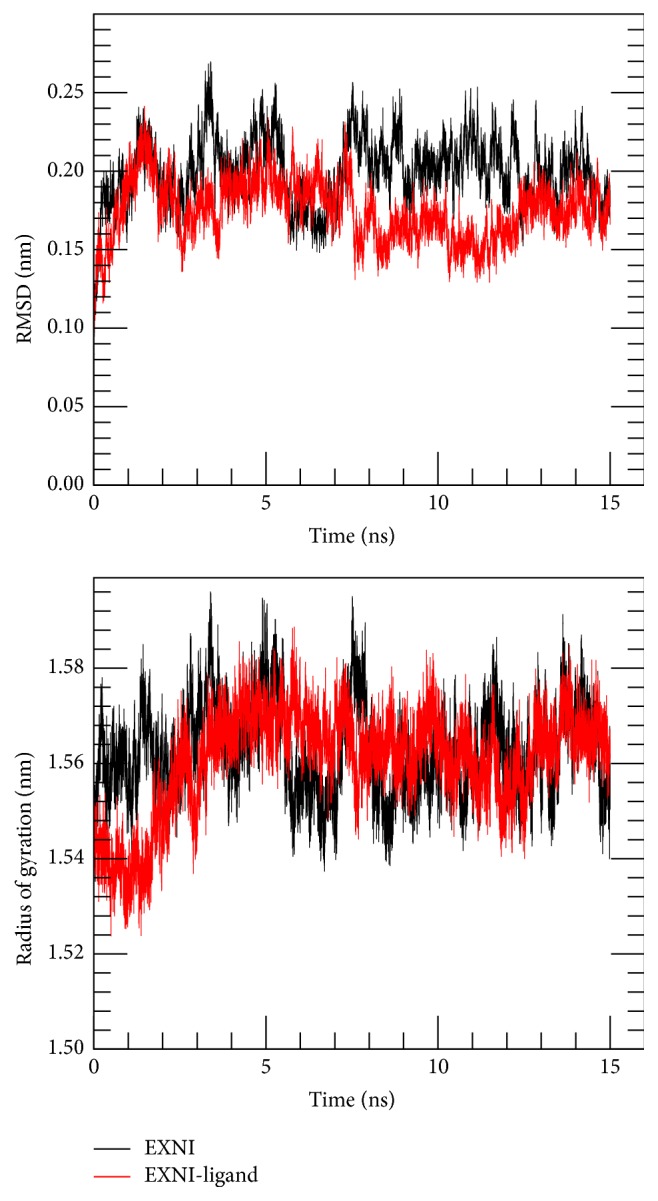
Root mean square deviations (RMSD) of the backbone and radius of gyration (Rg) of the protein from 15 ns long MD trajectory.

The top pane of Figure 7 compares the root mean square deviation (RMSD) of the EXNI backbone of bare protein and the protein-ligand (EXNI-xylan) complex system separately. Both systems indicate stable structures by maintaining a steady RMSD of about 0.2 nm with the simulation time. The bottom pane of Figure 7 gives the variation of radius of gyration (Rg) as a function of simulation time which indicates the compactness of the protein. As seen in the figure, Rg of both systems were maintained approximately the same value with minimum deviations. These results suggest that EXNI maintains its tertiary structure even after forming a complex with the ligand (xylan).

**Figure 8:**
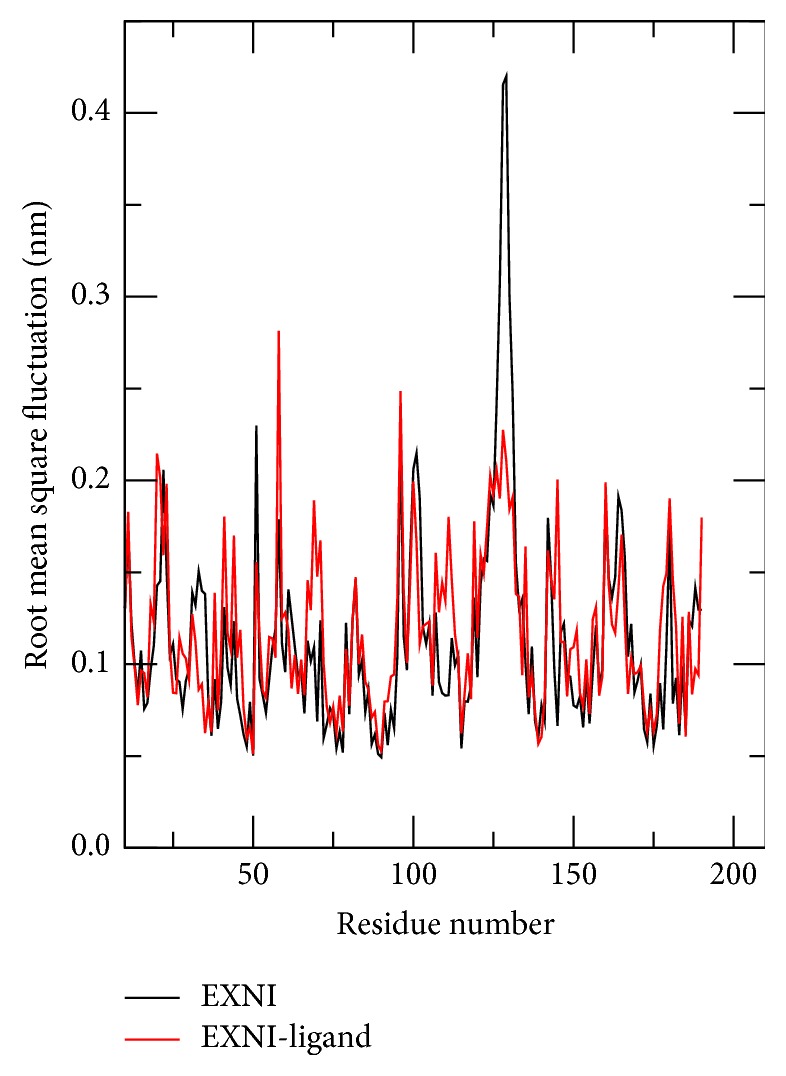
Root mean square fluctuation (RMSF) of the residues in the protein over 15 ns long MD trajectory.

The root mean square fluctuations (RMSF) of amino acid residues of EXNI (Figure 8) represent the stability of 3D structures for both systems of bare protein and protein-ligand complex. Most of the fluctuations are concentrated in 96, 100, and 128 amino acid residues in both systems. Significant fluctuations of amino acid residues have been observed in 126–131 regions in the free protein and in 58 and 98 positions in the protein-ligand complex. Further, none of the high fluctuating residues of the protein of the complex were in the predicted active site. Thus it can be postulated that EXNI can initiate its catalytic activity with xylan (ligand) via either retention or inversion mechanisms.

Five probable models were obtained in homology modeling from the MODELLER 9.13 software and they were ranked according to their normalized Discrete Optimized Protein Energy (zDOPE) and GA341 score. The model comprising the best scores was selected as the theoretical model for EXNI protein.

## 4. Conclusion

EXNI gene was successfully characterized, cloned, and expressed from locally isolated* T. virens* in* P. stipitis*. Three potential N-linked glycosylation sites were identified in* EXN*I. The increase in molecular weight observed from SDS-PAGE analysis of* EXN*I compared to the theoretically calculated value can be attributed to hyperglycosylation. Recombinant* P. stipitis *containing* EXN*I has the potential to degrade xylan the major component of hemicellulose fraction in plant biomass. Moreover it can be utilized to produce bioethanol via the combinatory simultaneous action with* XYL* for the hydrolysis and fermentation of xylan into ethanol.

Molecular docking studies provided major information on ligand binding domain and the active site residues of the protein. MD simulation results indicated almost similar and higher stability of EXNI in the form of protein-ligand complex and also in the free form of the protein. Enzyme-substrate association was steadily maintained by three hydrogen bonds in the ligand binding domain of EXNI. The predicted model of EXNI is realistic and therefore it provides more information of EXNI-xylan interaction for the designing of mutagenic experiments aimed at improving the catalytic action.

## Figures and Tables

**Table 1:** Statistics of the 3D model of EXNI from the Ramachandran plot.

Ramachandran plot statistics	EXNI	
Amino acid in most favoured regions	143	92.3%
Amino acid in additional allowed regions	11	7.1%
Amino acid in generously allowed regions	1	0.6%
Amino acid in disallowed regions	0	0.0%
Number of nonglycine and nonproline residues	155	
Number of end residues	2	
Number of glycine residues	27	
Number of proline residues	6	
Total number of residues	190	

**Table 2:** Detailed information of H bonds formed between ligand and the protein (A-Acceptor and D-Donor).

Residue	Amino acid	Distance H-A	Distance D-A	Donor angle	Protein donor	Side chain
16	SER	2.79	3.39	119.03	No	Yes
173	ILE	2.84	3.47	157.74	Yes	No
77	TYR	3.14	2.94	69.35	Yes	Yes
